# Argentatin B Inhibits Proliferation of Prostate and Colon Cancer Cells by Inducing Cell Senescence

**DOI:** 10.3390/molecules201219757

**Published:** 2015-11-27

**Authors:** Ela Alcántara-Flores, Alicia Enriqueta Brechú-Franco, Patricia García-López, Leticia Rocha-Zavaleta, Rebeca López-Marure, Mariano Martínez-Vázquez

**Affiliations:** 1 Instituto de Química, Departamento de Productos Naturales, Universidad Nacional Autónoma de México, Circuito Exterior, Ciudad Universitaria, Coyoacán, C.P. 04510, México D.F., Mexico; elaalfl@hotmail.com; 2 Posgrado en Ciencias Biológicas, Universidad Nacional Autónoma de México, Ciudad Universitaria 3000, Coyoacán, CP. 04510, México D.F., Mexico; 3 Facultad de Ciencias, Departamento de Ecología y Recursos Naturales, Universidad Nacional Autónoma de México, Coyoacán, C.P. 04510, México D.F., Mexico; aliciae@ciencias.unam.mx; 4 Instituto Nacional de Cancerología, Subdirección de Investigación Básica, Tlalpan, C.P. 14080, México D.F., Mexico; pgarcia_lopez@yahoo.com.mx; 5 Instituto de Investigaciones Biomédicas, Departamento de Biología Molecular y Biotecnología, Universidad Nacional Autónoma de México, Circuito Escolar s/n, Coyoacán, C.P. 04510, México, D.F., Mexico; lrochaz@biomedicas.unam.mx; 6 Instituto Nacional de Cardiología “Ignacio Chávez”, Departamento de Biología Celular, Juan Badiano No. 1, Colonia Sección 16, Tlalpan, C.P. 14080, México D.F., Mexico; rlmarure@yahoo.com.mx

**Keywords:** argentatin B, colon cancer, prostate cancer, cell senescence, xenografts

## Abstract

Argentatin B has been shown to inhibit the growth of colon HCT-15, and prostate PC-3 cancer cells. However, the mechanism by which argentatin B inhibits cell proliferation is still unknown. We aimed to investigate the mechanism by which argentatin B inhibits cell proliferation. The cell cycle was studied by flow cytometry. Apoptosis was evaluated by Annexin-V-Fluos, and Hoechst 33342 dye staining. Cell senescence was evaluated by proliferation tests, and staining for SA-β-galactosidase. Senescence-related proteins (PCNA, p21, and p27) were analyzed by Western blotting. Potential toxicity of argentatin B was evaluated in CD-1 mice. Its effect on tumor growth was tested in a HCT-15 and PC-3 xenograft model. Argentatin B induced an increment of cells in sub G1, but did not produce apoptosis. Proliferation of both cell lines was inhibited by argentatin B. Forty-three percent HCT-15, and 66% PC-3 cells showed positive SA-β-galactosidase staining. The expression of PCNA was decreased, p21 expression was increased in both cell lines, but p27 expression increased only in PC-3 cells after treatment. Administration of argentatin B to healthy mice did not produce treatment-associated pathologies. However, it restricted the growth of HCT-15 and PC-3 tumors. These results indicate that treatment with argentatin B induces cell senescence.

## 1. Introduction

It is known that natural compounds have been an important source of several clinically useful anti-cancer agents. In 2012, approximately 42% of compounds in clinical trials as antitumor agents were natural products, compounds derived from secondary metabolites, or designed from a natural product pharmacophore [1]. Some of the most important natural anti-cancer compounds include vinblastine, vincristine, camptothecin derivatives topotecan and irinotecan, etoposide, derived from epipodophyllotoxin, and paclitaxel. Furthermore, there are several kinds of natural products that are in preclinical development. At present, intensive efforts to identify new natural antitumor agents are in progress [2]. One important group of natural products is constituted of triterpenes. Some of these secondary metabolites have shown important anti-inflammatory and antitumor activities [3]. It has been reported that some triterpenoids inhibit proliferation of tumor cells [4] by different mechanisms. They can interfere with DNA replication by inhibiting DNA polymerase [5], topoisomerase I [6], and topoisomerase II [7]. Other cytotoxic triterpenoids induce modifications of the cytoskeleton by depolymerizing actin fibers [8], or changing the expression of cytoskeleton proteins [9]. Argentatin B is a cycloartane-type triterpene derived from the resin of *Parthenium argentatum* Gray (guayule), an endemic plant from Northern Mexico and Southwestern USA. This species has been used as a source of natural rubber [10,11,12]. In a former work, we demonstrated that it is a non-competitive inhibitor of 3H-estradiol binding to receptors on human, hormone-dependent breast tumors [13]. We also found that argentatin B inhibits, in a dose-dependent manner, the edema induced by the tumor promoter 12-*O*-tetradecanoylphorbol-13-acetate (TPA). Furthermore, we observed that argentatin B can inhibit the growth of human colon carcinoma (HCT-15) and human prostate cancer (PC-3) cell lines [14]. Interestingly, argentatin B did not produce cytotoxic or genotoxic effects on lymphocytes from healthy human donors [15]. However, the mechanism by which argentatin B inhibits cell proliferation is still unknown. Thus, in the present work we aimed to investigate the mechanism by which argentatin B mediates inhibition of tumor cells proliferation. We also aimed to evaluate the effect of argentatin B in healthy mice, and on a human xenograft model, using the PC-3 and HCT-15 cell lines.

## 2. Results and Discussion

### 2.1. Isolation of Argentatin B

**Figure 1:**
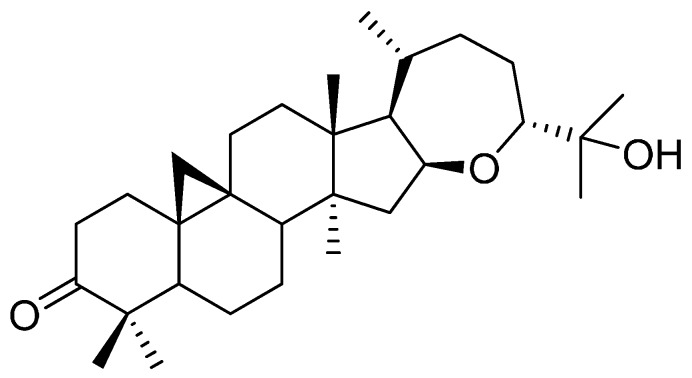
Chemical structure of argentatin B (16β*,* 24*R*-16,24-epoxi-25-hidroxicicloartan-3-one).

Argentatin B was isolated from *P. argetatum* as previously reported and purified at 99% by conventional procedures [10,11]. It was identified by comparison of physical and spectroscopic constants (melting point, ^1^H, and ^13^C Nuclear Magnetic Resonance) with those reported in the literature [12]. The structure of argentatin B, (16β,24*R*-16,24-epoxi-25-hidroxicicloartan-3-one), is shown in Figure 1.

### 2.2. Argentatin B Induces Changes on Cell-Cycle Progression

**Figure 2:**
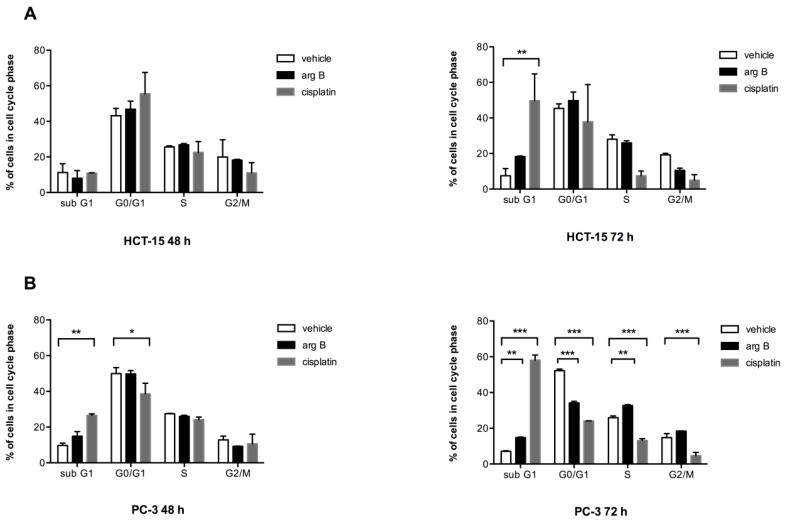
Effect of argentatin B on cell cycle progression. HCT-15 (**A**) and PC-3 (**B**) cells were incubated with 24 μM or 34 μM argentatin B, respectively, for 48 and 72 h. As a positive control cells were treated with cisplatin 14 μM. Negative control cultures received the carrier solvent (0.2% DMSO). The cells were stained with propidium iodide, and the cell cycle distribution was analyzed by flow cytometry using the BD CellQuest Pro Software. Data represent the average of three independent assays. Error bars indicate the standard error of the mean. * *p* < 0.05, ** *p* < 0.001, and *** *p* < 0.0001 *vs.* vehicle (one-way ANOVA test, and Tukey-Kramer post-test).

We first determined the concentrations of argentatin B and cisplatin leading to 50% inhibition of cell proliferation (IC_50_) for each cell line. Argentatin B IC_50_ was 24 μM for HCT-15 and 34 μM for PC-3. Meanwhile, cisplatin IC_50_ was determined to be 14 μM for both cell lines. To investigate whether argentatin B inhibits DNA replication we analyzed cell-cycle progression of cells treated with the determined argentatin B IC_50_. As a control, cells treated with the cisplatin IC_50_ were analyzed. As seen in Figure 2A, no significant changes in cell cycle phases were observed in HCT-15 cells after 48 h of treatment. However, cells treated with argentatin B for 72 h showed a tendency to increase the proportion of cells in sub G1, along with a reduction of the number of cells in G2/M, but the proportion of cells in S phase remained unchanged. As expected, 72 h treatment with cisplatin produced a significant increase of the proportion of cells in sub G1 phase. It also reduced the proportion of cells in S and G2/M phases, although this reduction was not significant. On the other hand, changes in PC-3 cell cycle after 48 h were only observed when the cells were incubated with cisplatin (Figure 2B). Nevertheless, treatment with argentatin B for 72 h induced a significant increment in the proportion of cells in sub G1, along with a reduction of cells in G0/G1. In addition an unexpectedly, marginally significant increase of cells in S phase was observed. Again, the number of cells in G2/M phase was not disturbed by argentatin B. In contrast, cisplatin produced a highly significant increment of cells in sub G1, along with the reduction of cells in G0/G1, S, and G2/M phases (Figure 2B). These observations seem to suggest that argentatin B does not inhibit DNA replication, neither has a negative effect on cell mitosis.

### 2.3. Argentatin B Inhibits Cell Proliferation by Inducing Cell Senescence

**Figure 3:**
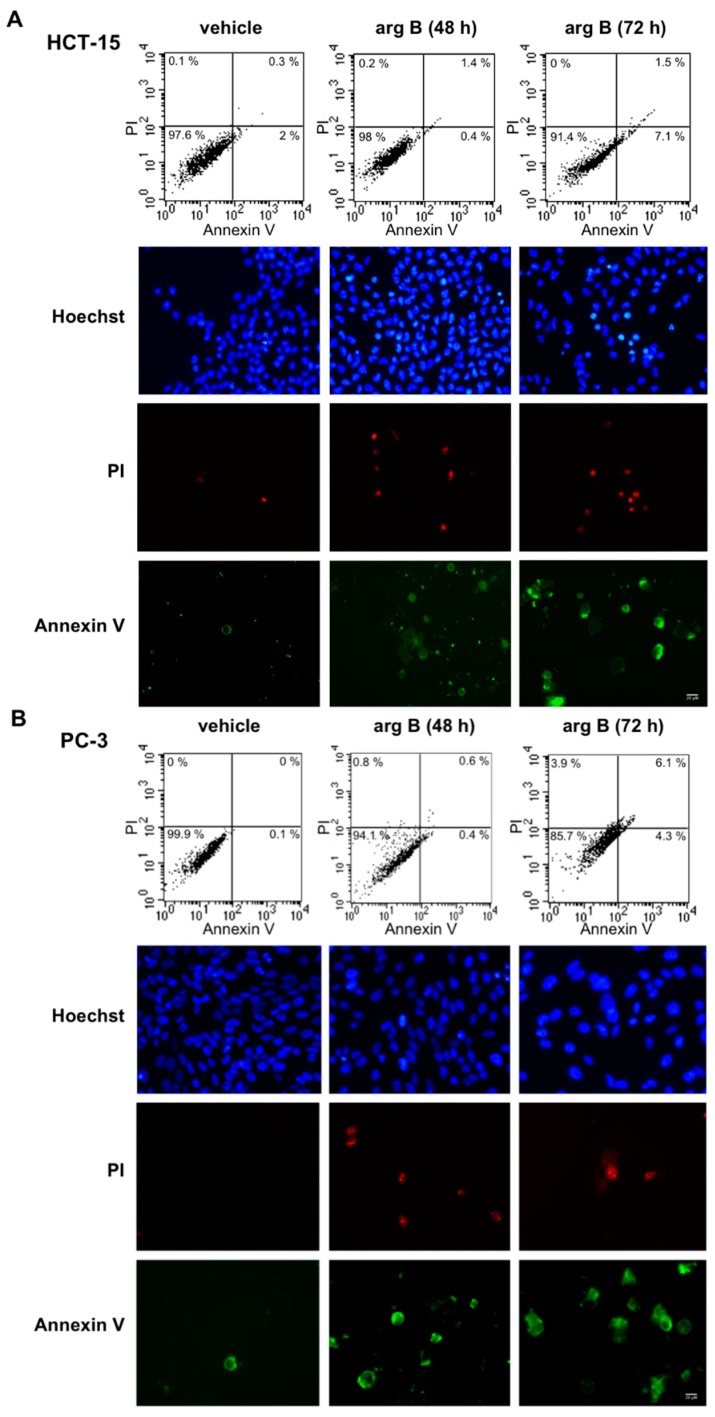
Effect of argentatin B on cell death. HCT-15 (**A**); and PC-3 (**B**) cells were incubated with argentatin B (arg B) for 48 h and 72 h. Cell death was analyzed by labelling with Annexin V and Propidum Iodide (PI). The number of apoptotic and necrotic cells was evaluated by flow cytometry (upper panel). The proportion of viable cells, showing negative annexin and PI staining is depicted in the left lower quadrant. Apoptotic cells, positive annexin, are shown in the right lower quadrant. Necrotic cells, positive annexin and PI staining, are presented in the right upper quadrant. Results are representative figures from three independent tests. Cells stained with Annexin, PI, and Hoechst were also analyzed by fluorescence microscopy (lower panel). Figures are representative micrographs from three independent experiments.

**Figure 4:**
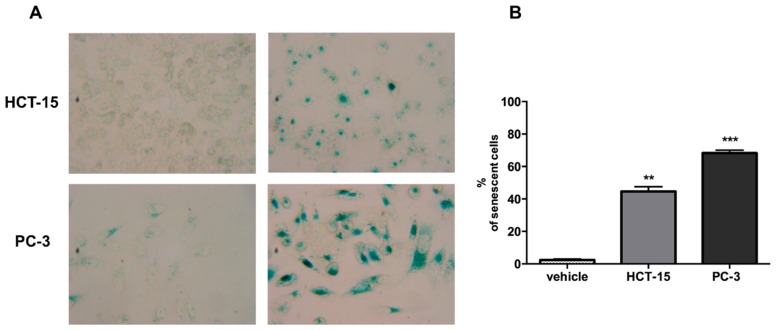
Argentatin B induces cell senescence at 72 h. (**A**) Representative micrographs of HCT-15 and PC-3 treated with argentatin B or vehicle (Magnification, ×40); (**B**) SA-β-gal-positive cells were evaluated by counting more than 100 cells for each treatment. Values presented are the mean of three independent experiments. Error bars indicate the standard error of the mean. ** *p* < 0.001, and *** *p* < 0.0001 *vs.* vehicle (one-way ANOVA test, and Tukey-Kramer post-test)

Since argentatin B induced an increase of cells in sub G1, we next investigated whether argentatin B can induce apoptotic cell death. After incubation of HCT-15 and PC-3 cells with argentatin B for 48 and 72 h, cell death was evaluated by staining with annexin V and propidium iodide. As shown in Figure 3, argentatin B induced a modest increment of apoptotic (7.1%), and necrotic cells (1.5%) after 72 h incubation. Likewise, after 72 h incubation, a slight increment of apoptotic (4.3%), and necrotic (6.1%) PC-3 cells was observed (Figure 3). These observations indicate that argentatin B is unable to induce a cytotoxic effect. However, we had previously demonstrated that argentatin B inhibits cell proliferation. Therefore, in an attempt to explain the observation mentioned above, we tested the cells for the presence of senescence. As seen in Figure 4A, after incubation with argentatin B for 72 h, both cell lines exhibited phenotypic changes that resemble those observed in cells undergoing senescence, such as flattened morphology and enlarged cell size. When tested for senescence associated-β-galactosidase activity, a proportion of 43% HCT-15, and 66% PC-3 cells showed a positive staining, compared with 2% of untreated controls. These findings suggest that argentatin B inhibits cell proliferation by inducing senescence.

**Figure 5:**
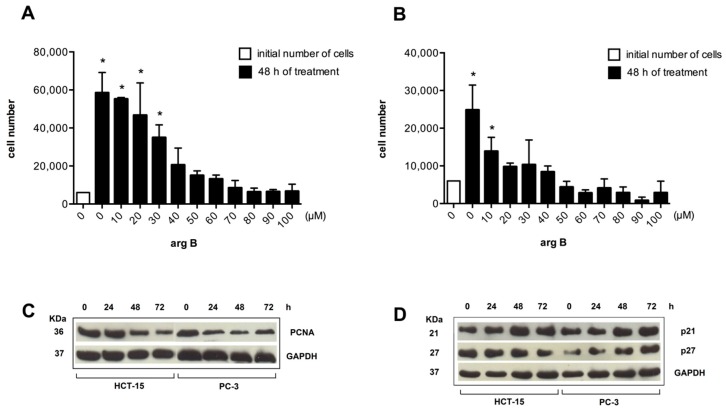
Argentatin B inhibits cell proliferation and increases expression of senescence-associated proteins. HCT-15 (**A**), and PC-3 (**B**) cells were treated with the indicated concentrations of argentatin B for 48 h. Cell numbers were evaluated by using the colorimetric MTT assay. Values presented are the mean of three independent experiments. Error bars indicate the standard error of the mean. * *p* < 0.05, *vs.* the number of cells seeded at the beginning of the experiment (initial number of cells) (one-way ANOVA test, and Tukey-Kramer post-test); (**C**) Western blot analysis of the expression of the proliferation marker PCNA. HCT-15, and PC-3 were incubated with 24 μM or 34 μM argentatin B, respectively for 24, 48, and 72 h. Detection of GAPDH was included as an internal control; (**D**) Western blot analysis of the expression of negative cell cycle regulators p21 and p27 in response to argentatin. HCT-15 and PC-3 cells were incubated with 24 μM or 34 μM argentatin B, respectively for 24, 48, and 72 h. Detection of GAPDH was included as an internal control.

It is known that the main characteristic of senescent cells is the inhibition of proliferation. PCNA expression is a hallmark of cell division. Thus, we analyzed the effect of increasing concentrations of argentatin B on cell proliferation, and its effect on the expression of PCNA. As shown in Figure 5, argentatin B induced a reduction of cell proliferation in a dose-dependent manner in both, HCT-15 (Figure 5A), and PC-3 (Figure 5B) cells. A significant reduction in the number of cells was observed from 30 to 100 μM of argentatin B for HCT-15, and from 10 to 100 μM of argentatin B for PC-3 cells. However, they were never significantly smaller than the initial number of cells seeded, supporting the observation that argentatin B induces the inhibition of proliferation rather than cell death. Accordingly, Western blot analysis of PCNA showed that the expression of this cell division-associated protein decreased when the cells were incubated with argentatin B (Figure 5C). It is accepted that senescence is promoted by a number of anti-proliferative mechanisms. Classical negative cell cycle regulators, such as p21, and p27, have been associated with the senescent phenotype. Therefore, we next analyzed the expression of p21 and p27 in cell cultures treated with argentatin B. HCT-15 and PC-3 cells were incubated with 24 μM or 34 μM argentatin B, respectively. The expression of p21 and p27 was evaluated at 24, 48, and 72 h by Western blotting. As seen in Figure 5D, treatment with argentatin B induced an increment of both, p21 and p27 in PC-3 cells after 48 h incubation, and it was persistent for at least 72 h after treatment. In the case of HCT-15, an increment of p21 expression was observed after 48 h treatment and it was persistent until 72 h of argentatin B treatment. In contrast, the level of p27 was not changed in HCT-15 after 24–48 h argentatin B treatment, and a reduction of p27 expression was observed at 72 h of treatment. Taken together, these results indicate that treatment with argentatin B induces the cells to undergo senescence.

### 2.4. Argentatin B Reduces Tumor Growth in Vivo

**Figure 6:**
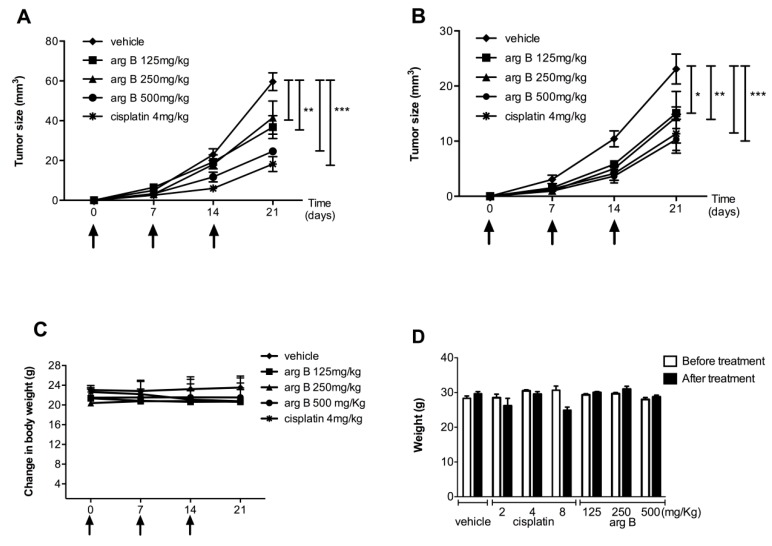
Evaluation of effects of argentatin B *in vivo*. Antitumor activity of argentatin B was evaluated in *nu/nu* mice implanted with HCT-15 (**A**) and PC3 (**B**) cells. Mice received the indicated concentrations of argentatin B, cisplatin or sesame oil (vehicle) at days 0, 7, and 14. Each point represents the average ± SEM of six animals. Significant differences of * *p* < 0.05, ** *p* < 0.001, and *** *p* < 0.0001 *vs.* vehicle are shown (one-way ANOVA test, and Tukey-Kramer post-test); (**C**) Evaluation of body weight change in *nu/nu* mice; (**D**) The toxicity of argentatin B was tested in groups of three CD-1 mice. The indicated doses of argentatin B, cisplatin or vehicle were administered intraperitoneally every week. The weight of the mice was recorded every other day. Error bars indicate the standard error of the mean.

To further examine the antiproliferative effect of argentatin B, *in vivo* xenografts using HCT-15 and PC-3 cells were established in mice. Animals were treated with three different concentrations of argentatin B or vehicle (sesame oil). Treatment with cisplatin was used as a positive control. As shown in Figure 6, the growth of HCT-15 tumors was significantly reduced after treatment with 125 mg/kg (*p* < 0.001), 250 mg/kg (*p* < 0.001), and 500 mg/kg argentatin B (*p* < 0.0001), by day 21 (Figure 6A). As expected, growing of HCT-15 tumors was also inhibited by cisplatin (Figure 6A). As seen in Figure 2B, growing of PC-3 tumors was significantly reduced by treatment with cisplatin (*p* < 0.0001), 500 mg/kg (*p* < 0.0001), 250 mg/kg (*p* < 0.001), and 125 mg/kg argentatin B (*p* < 0.05) at the end of the experiment (day 21). The toxicity of treatments is shown in the Figure 6C, no change in weight was observed, indicating no systemic toxicity with any of the treatments. Our results suggest that argentatin B restricts HCT-15 and PC-3 cells proliferation *in vivo* as efficiently as cisplatin. We next examined the potential systemic toxicity of argentatin B in healthy mice. All animals survived the treatment with argentatin B. No significant differences in general appearance, depression of activity, respiratory difficulty, abnormal aggressive behavior, and in mean daily food and water consumption between the experimental groups and the untreated controls were observed. Histopathological analysis showed no evidence of treatment-related pathology. Besides, no significant differences in body weight after and before treatment were observed (Figure 6D), suggesting that argentatin B is not toxic at the concentrations tested.

### 2.5. Discussion

Here we have presented evidence that argentatin B inhibits proliferation of HCT-15 and PC-3 cells by inducing senescence. The cytotoxic effect of some cycloartane-type triterpenoids has been associated with a strong pro-apoptotic activity [16]. Some cycloartane-type triterpenoids have been isolated and tested against cancer cells. Their cytotoxic effect has been reported to be mediated by some potential mechanisms. There is evidence suggesting that triterpenoids inhibit cell proliferation by interfering with DNA replication [5,6,7]. Interestingly, a cycloartane-type triterpenoid isolated from *Commiphora opobalsamum* showed a moderate antiproliferative effect on human prostate cancer cells. However, it was able to inhibit the expression of androgen receptors in the cells [17], suggesting that triterpenoids may modulate some mechanisms involved in the regulation of cell proliferation. Tian *et al.* [18] reported that schisandrolic and isoschisandrolic acids exerted their cytotoxic effect via G0/G1 arrest and subsequent apoptosis. There is evidence showing that cycloartane-type triterpenoids may induce both, cytostatic and cytotoxic effects, increasing the number of cells in sub G1, and arresting cells in S and G2/M phases [19]. Accordingly, we observed that treatment with argentatin B induced a significant increment in the proportion of cells in sub G1. Accumulation of cells in sub G1, has also been documented to occur as a cellular response to powerful reactive oxygen species, causing oxidative stress that leads the cell to develop a condition of senescence [20].

It has been shown that tumor growth-inhibitory effects exhibited by natural compounds, such as resveratrol, can be attributed to the induction of cell senescence [21,22]. Here we observed that cells treated with argentatin B presented phenotypic changes that resembled those detected in cells undergoing senescence, such as flattened morphology and enlarged cell size. In fact, only after 48 h these changes are evident and at 72 h treatment 66% PC-3, and 43% HCT-15 cells were positive for senescence associated-β-galactosidase activity. Cellular senescence is a complex, multifactorial condition of arrested growth. Senescence-associated signaling pathways activate important cell cycle negative regulators, like p21 which is a strong inhibitor of cyclin-dependent kinases [23], and p27 which is a negative regulator of G1 progression [24]. Accordingly, the expression of p27 was elevated in PC-3 cells as a response to argentatin B treatment. However, the level of p27 in HCT-15 remained unchanged during the first 48 h of treatment, and decreased at 72 h. In contrast, a clear elevation of p21 levels was detected in both PC-3, and HCT-15 cells. In accordance with our results, previous reports have demonstrated that inhibitors of proliferation, such as adamantane derivatives [25], and celecoxib [26], consistently induce an increment of p21 expression in different colon cancer-derived cell lines. Nevertheless, adamadate derivative DPA produced no change, or even a reduction of p27 in colon cancer-derived Colo 205 and HT-29 cell lines [25], while celecoxib induced a decrease of p27 levels at short times incubation (0–5 to 8 h), followed by an increasing expression of p27 at 24 h in HCT-15, HCT-29, and Caco-2 cell lines [26].

Our results showed that argentatin B inhibited cell proliferation, decreased the expression of PCNA (proliferation-related marker), and also induced an increment of p21 expression in HCT-15 and PC-3 cells. It will very interesting to determine if argentatin B affects other proteins involved with cellular senescence such as p16, p53 or proinflammatory molecules.

Since this protein has been associated with a full senescence state, these observations provide evidence to support the fact that argentatin B induces senescence. Cellular senescence is currently considered an important target for cancer treatment, inasmuch as it leads to an irreversible obstruction of the cellular division capacity. Moreover, tumor cells activate senescence mechanisms as a response to chemotherapy and radiotherapy [27].

Cellular senescence induced by stress is known as premature senescence [27]. It occurs within a week of exposure to sub lethal stresses. Senescent PC-3 and HCT-15 cells were observed three days after argentatin B treatment, suggesting the induction of premature senescence. There is evidence showing that other triterpenoids can induce senescence. For instance, Chang *et al.* [28] reported that ganoderiol F, a tetracyclic triterpene isolated from *Ganoderma amboinense*, induces senescence after 18 days of continuous treatment of HepG2. Similarly, administration of 20–50 μM resveratrol has been reported to produce senescence in lung cancer cells after 10 to 12 days incubation [21]. In sharp contrast, concentrations of 24 and 34 μM argentatin B can induce senescence in confluent cell cultures in only 48–72 h. Interestingly, commonly used chemotherapeutic drugs, such as cisplatin, doxorubicin, etoposide and other topoisomerase inhibitors are capable of inducing senescence of cancer cells when they used in very low concentrations [29,30].

In the present study we observed that argentatin B can inhibit the growth of human colon carcinoma (HCT-15, IC_50_ 24.14 ± 5.58) and human prostate cancer (PC-3, IC_50_ 34.14 ± 3.71 μM) cell lines. We know that triterpenes such ursolic acid and acetyl-boswellic acid with low cytotoxic activities showed an effective antitumor effect in mice xenograft model. With this in mind, we decide to evaluate the antitumor activity of argentatin B in a xenograft mice model using PC-3 and HCT-15 human cell lines.

Our results suggest that argentatin B restricts HCT-15 and PC-3 cells proliferation *in vivo* as efficiently as cisplatin. Frequently, *in vivo* testing of new antitumor agents is carried out using daily administrations of the drug for 15-days periods. We also demonstrated that three weekly administrations of argentatin-B were enough to induce a significant decrease of tumor growth, and produced no toxic effects on the experimental animals. It has become clear that tumor cells can undergo senescence in response to chemotherapy. Mitotic arrest is normally not tolerated by cells, and it is resolved by cell death. Thus, cytostatic activity can be followed by cell death. In fact, many cytotoxic agents are primarily cytostatic [31]. Thus, stimulation of this response is nowadays considered a rational approach to cancer treatment. In this work, we have shown that argentatin B can inhibit cell proliferation by inducing cellular senescence. However, the demonstration of cytostasis or cytotoxicity depends on experimental conditions, so it would be important to test argentatin B under a wide range of doses and times schedules, both *in vitro* and *in vivo* to evaluate its potential pharmaceutical usefulness.

## 3. Experimental Section

### 3.1. Drugs and Reagents

Roswell Park Memorial Institute medium (RPMI-1640) was obtained from Caisson Laboratories INC, USA. FBS (fetal bovine serum), EDTA, amphotericin B and l-glutamine were obtained from Gibco, BRL (Grand Island, NY, USA). Trypsin, DMSO, Propidium iodide, Hoechst 33342 and cisplatin (*cis*-Diammineplatinum (II) dicloride) were obtained from Sigma-Aldrich (St. Louis, MO, USA). Annexin-V-Fluos staining kit was from Roche Diagnostics (GmbH, Mannheim, Germany), and Senescence β-Galactosidase Staining Kit was obtained from Cell Signaling Technology (Denver, MA, USA). High-quality water employed to prepare solutions was obtained through a Milli-Q Reagent Water System (Continental Water Systems; El Paso, TX, USA).

#### Solutions

Stock solutions of cisplatin were prepared in saline solution (1 mg/mL) for *in vivo* studies, and in DMSO (20 mM) for *in vitro* assays. Argentatin B (40 mg/mL) was dissolved in extra virgin sesame oil. Stock solutions of argentatin B (20 mM) were prepared in DMSO for *in vitro* assays, and stored at −20 °C.

### 3.2. Isolation of Argentatin B

Argentatin B was isolated from *Parthenium argentatum* as previously reported [10,11].

### 3.3. Cell Cultures

The human colon cancer HCT-15 and prostate cancer PC3 cell lines used in this study were purchased from ATCC (HCT-15 CCL-225™ and PC-3 CRL-1435™) (Rockville, MD, USA). The cells were routinely maintained as a monolayer in RPMI supplemented with 10% inactivated FBS, 250 U/mL streptomycin sulfate, 100 IU/mL penicillin, 0.25 mg/mL amphotericin B and 2 mM l-glutamine, and incubated at 37 °C in a 5% CO_2_ atmosphere at high humidity. Cells were harvested with 0.025% Trypsin and 1 mM EDTA. 5 × 10^3^ cells were seeded in 96-well plates, and incubated for 2 h. The cells were treated with increasing concentrations (1 to 100 μM) of argentatin B or 14 μM cisplatin, diluted in 0.2% DMSO, for 48 h at 37 °C. After discarding the medium, the cells were fixed by adding 50 μL of cold, 50% trichloroacetic acid, and then incubated with 0.4% sulforhodamine (SRB, Sigma Chemicals) for 30 min at room temperature. After washing three times with 1% acetic acid the plates were air-dried, and the protein-bound SRB was dissolved with TRIZMA base 10 mM. Optical density was measured on an ELISA plate reader (EL ×800, BioTek, Winooski, VT, USA) at 515 nm. The argentatin B, or cisplatin concentration producing a 50% inhibition of cell proliferation (IC_50_) was determined for each cell lines.

#### 3.3.1. Flow Cytometric Detection of Cell Cycle

Cell cycle was determined by flowcytometric assays. 1.5 × 10^5^ cells were seeded in 6-well plates and incubated with argentatin B, control cultures received the carrier solvent (0.2% DMSO) for 48 and 72 h. Cells were harvested by trypsinization, washed with PBS and resuspended in 70% ethanol at 4 °C for at least 10 min. Thereafter, cells were washed with PBS and incubated for 60 min in a solution containing 50 U/mL RNAse in PBS at 37 °C. The cells were washed with PBS and stained with a solution of 20 μL/mL propidium iodide in PBS (50 μg/mL), for 2 min. The cell cycle distribution was analyzed with a flow cytometer FACSCalibur (Becton Dickinson, Franklin Lakes, NJ, USA). 10,000 cells were analyzed with the BD CellQuest Pro Software (Becton Dickinson).

#### 3.3.2. Flow Cytometric Detection of Apoptotic Cells

Treated and control, 48 and 72 h, HCT-15 and PC-3 cells were harvested by trypsin release and washed twice with ice-cold PBS. Apoptotic cell death was determined using the Annexin-V-Fluos staining kit, according to the manufacturer’s instructions. Cell samples were analyzed on the flowcytometer FACSCalibur (Becton Dickinson). 10,000 cells were analyzed with BD CellQuest Pro Software.

#### 3.3.3. Detection of apoptotic cells by HOECHST 33342 Dye

Cells were seeded in 24-well plates and incubated for 48 and 72 h with argentatin B. Thereafter, cells were fixed in 3.7% formaldehyde solution for 10 min and washed for 10 min with PBS, subsequently the cells were washed with deionized water and stained with Hoechst 33,342 diluted 1:1000 in PBS for 15 min at 37 °C. Cells were then observed in a microscope Nikon Optiphot-2. Cells with condensed and fragmented nuclei were judged to be apoptotic.

#### 3.3.4. Cytochemical Staining for SA-β-Galactosidase

7.5 × 10^3^ cells were seeded in 24-well plates and incubated with argentatin B. Cytochemical staining for SA-β-galactosidase was performed using a Senescence β-Galactosidase Staining Kit at pH 6.0. Cells were rinsed with PBS, and the percentages of SA-β-galactosidase positive (blue) cells were determined after scoring 300 cells for each sample using a bright-field microscope Nikon Optiphot-2. All the experiments were repeated three times.

#### 3.3.5. Proliferation Assay and Western Blot Analysis

To evaluate cell proliferation, 5 × 10^3^ HCT-15 or PC-3 cells were seeded in 96-well plates, and incubated in the presence of 0, 10, 20, 30, 40, 50, 60, 70, 80, 90, and 100 μM argentatin B diluted in 0.2% DMSO for 48 h. Proliferation was evaluated by the colorimetric MTT assay. For Western blot analysis the cells were resuspended in lysis buffer (50 mM Tris-HCl, pH 7.4; 150 mM NaCl; 1 mM EDTA; 1% NP40; 0.25% sodium deoxycholate), containing 100 μL/mL complete protease inhibitors cocktail (Roche Applied Science, Mannheim, Germany) and 10 μL/mL phosphatase inhibitors (Sigma-Aldrich). Total protein content was determined using the DC protein assay kit (BioRad Laboratories, Hercules, CA, USA). A total of 30 μg of protein was resolved by 10% SDS-PAGE and transferred onto polyvinylidene fluoride (PVDF) membranes (Millipore, Billerica, MA, USA). Membranes were incubated at 4 °C, overnight with specific antibodies diluted 1:1000 and then washed and incubated with the appropriate horseradish peroxidase-conjugated secondary antibodies diluted 1:5000 (Zymed Laboratories, Invitrogen Life Technologies, Carlsbad, CA, USA). Primary antibodies used were, rabbit-monoclonal anti-human PCNA (GeneTex Inc., Irving, CA, USA, USA), mouse-monoclonal anti-human p21, and mouse-monoclonal anti-human p27 (both from Santa Cruz Biotechnology, Santa Cruz, CA, USA, USA). Proteins were detected by chemiluminescence using the Amersham ECL plus Western Blotting Detection System (GE Healthcare Bio-Sciences, Piscataway, NJ, USA). As an internal control, a rabbit anti-GAPDH (GeneTex Inc.) was included.

### 3.4. Tumor Xenografts

Male, 6–8-week-old *nu*/*nu* mice were provided by the Animal House of the National Institute for Nutrition “Salvador Zubirán” (México D.F., Mexico), they were kept in a pathogen-free environment and fed *ad livitum*. Experimental procedures were carried out in accordance with the Guidelines for Care and Use of Laboratory Animals of the National Cancerology Institute (México D.F., Mexico). Groups of six animals were implanted with 1.5 × 10^6^ HCT-15 and PC3 cells. Cells were inoculated subcutaneously in the right flank of the mice, once tumors had reached approximately 50 mm^3^, the animals were pair-matched into treatment and control groups and the treatments were initiated. Then, mice received either 125, 250 or 500 mg/kg argentatin B diluted in sesame oil at days 0, 7, and 14. Control animals received only the vehicle (sesame oil) administered intraperitoneally as negative control and cisplatin as a positive control at the dose of 4 mg/kg. Mice were weighed periodically. Tumor size was measured by using a calliper twice a week. Tumor volume was determined by using the following relation: V (mm^3^) = π/4 × [large diameter × (short diameter)^2^]. The relative tumor volume was calculated using the formula: (Volume on the evaluation day /volume on day 0) × 100. After each drug administration, mice were weighed and the tumor volume was calculated, as previously described (every three days). The experiment was conducted during twenty-one days, at the end of which time all animals were weighed and euthanized.

#### Evaluation of Argentatin B Toxicity

The toxicity of argentatin B was tested in groups of three female, 6-week-old CD-1 mice. Three doses of 125, 250, and 500 mg/kg argentatin B were administered intraperioteally once a week for 3 weeks, and compared with the effect of 2, 4, and 8 mg/kg of cisplatin. The weight and behavior of the mice was recorded every other day throughout the treatment and observation time (7 days after the last injection). Mice were sacrificed at day 22. The heart, lung, liver, kidney, intestine, spleen, stomach and ovaries were removed, fixed in 10% formalin, paraffin-embedded, and cut into 4 μm sections. The sections were deparaffinised in xylene, and rehydrated in graded concentrations of ethanol. Tissue sections were stained with Hematoxilin-Eosin for histopathologic analysis.

### 3.5. Data Analysis

Statistical analyses were performed using GraphPad Prism 6.0 software (GraphPad Software Inc., La Jolla, CA, USA). Comparisons between treated groups and untreated controls were carried out using one-way ANOVA test, and Tukey-Kramer post-test. Data were expressed as means ± SEM. The tests considered a basic significance level of *p* < 0.05.

## 4. Conclusions

The present work provides evidence that argentatin B inhibits the proliferation of HCT-5 and PC-3 cells, both *in vitro* and in a xenograft nude mice model. The efficiency of argentatin B was comparable with that of cisplatin. Argentatin B exhibited a rather weak apoptotic effect. However, it was able to inhibit cell proliferation by inducing cellular senescence. Induction of cell senescence has been considered a rational approach for the designing of new anti-tumor drugs. Thus, further research is in progress to evaluate argentatin B potential pharmacological value.
